# Low-flow assessment of current ECMO/ECCO_2_R rotary blood pumps and the potential effect on hemocompatibility

**DOI:** 10.1186/s13054-019-2622-3

**Published:** 2019-11-06

**Authors:** Sascha Gross-Hardt, Felix Hesselmann, Jutta Arens, Ulrich Steinseifer, Leen Vercaemst, Wolfram Windisch, Daniel Brodie, Christian Karagiannidis

**Affiliations:** 1 Department of Cardiovascular Engineering, Medical Faculty, Institute of Applied Medical Engineering, Helmholtz Institute, RWTH Aachen University, Aachen, Germany; 2 Department of Perfusion, University Hospital Gasthuisberg, Leuven, Belgium; 3 Department of Pneumology and Critical Care Medicine, Cologne-Merheim Hospital, ARDS and ECMO Center, Kliniken der Stadt Köln gGmbH, Witten/Herdecke University Hospital, Ostmerheimer Strasse 200, 51109 Cologne, Germany; 4 Center for Acute Respiratory Failure, Columbia University College of Physicians and Surgeons/New York-Presbyterian Hospital, New York, NY USA

**Keywords:** ARDS, ECMO, ECCO_2_R, ECLS, Centrifugal blood pumps

## Abstract

**Background:**

Extracorporeal carbon dioxide removal (ECCO_2_R) uses an extracorporeal circuit to directly remove carbon dioxide from the blood either in lieu of mechanical ventilation or in combination with it. While the potential benefits of the technology are leading to increasing use, there are very real risks associated with it. Several studies demonstrated major bleeding and clotting complications, often associated with hemolysis and poorer outcomes in patients receiving ECCO_2_R. A better understanding of the risks originating specifically from the rotary blood pump component of the circuit is urgently needed.

**Methods:**

High-resolution computational fluid dynamics was used to calculate the hemodynamics and hemocompatibility of three current rotary blood pumps for various pump flow rates.

**Results:**

The hydraulic efficiency dramatically decreases to 5–10% if operating at blood flow rates below 1 L/min, the pump internal flow recirculation rate increases 6–12-fold in these flow ranges, and adverse effects are increased due to multiple exposures to high shear stress. The deleterious consequences include a steep increase in hemolysis and destruction of platelets.

**Conclusions:**

The role of blood pumps in contributing to adverse effects at the lower blood flow rates used during ECCO_2_R is shown here to be significant. Current rotary blood pumps should be used with caution if operated at blood flow rates below 2 L/min, because of significant and high recirculation, shear stress, and hemolysis. There is a clear and urgent need to design dedicated blood pumps which are optimized for blood flow rates in the range of 0.5–1.5 L/min.

## Background

Extracorporeal life support (ECLS), which is comprised of extracorporeal membrane oxygenation (ECMO) and extracorporeal carbon dioxide removal (ECCO_2_R) [1], is an emerging technology in the field of respiratory medicine used for various indications, including the acute respiratory distress syndrome (ARDS) and acute exacerbations of chronic obstructive pulmonary disease (COPD), or as a bridge to lung transplantation [2–8]. Recently, the EOLIA trial demonstrated a survival benefit for patients treated with ECMO compared to standard of care in severe ARDS [9, 10]. However, extracorporeal systems have substantial side effects, in particular, bleeding or clotting may occur in many patients. The concept of ECCO_2_R has been proposed as a safer alternative to ECMO due to the lower blood flow rates and smaller cannulae used. However, greater safety has not been established, and recent studies demonstrate increased bleeding complications in patients treated with ECCO_2_R [5, 11].

Historically, ECCO_2_R systems were developed from renal replacement therapy (RRT) and driven by roller pumps [12–14] or from high-flow extracorporeal membrane oxygenation (ECMO) devices driven by rotary pumps; most of them were centrifugal blood pumps in recent years. Few systems were designed specifically for ECCO_2_R [15–17]. In patients with moderate-to-severe ARDS, the SUPERNOVA pilot trial recently demonstrated the feasibility of reducing the intensity of mechanical ventilation by applying ECCO_2_R, using three different extracorporeal devices with blood flow rates ranging from 300 to 1000 mL/min [2]. However, although all three systems were characterized as “ECCO_2_R” [18], there were distinct differences with regard to the efficacy of CO_2_ removal. Systems derived from RRT devices are limited in blood flow rates (usually up to 500 mL/min), whereas those that are derived from high-flow ECMO devices are, in general, not limited by the blood flow rate, but more by cannula (or catheter) size and membrane lung surface area. In daily clinical practice, systems operating at blood flow rates up to 500 mL/min remove CO_2_ on the order of 80 mL/min. This can be nearly doubled by doubling the blood flow rate, thereby accounting for approximately 50% of the CO_2_ production of an adult resting intensive care unit (ICU) patient [19–22]. Furthermore, ECMO therapy for neonatal and pediatric patients uses comparable blood flow rates with current rotary blood pumps.

Whereas the efficacy and technical determinants of ECCO_2_R for adults, or low-flow ECMO for neonatal and pediatric patients, are reasonably well characterized, studies have raised the issue of the safety of the treatment [5, 23]. Although the blood flow rates used in ECCO_2_R are lower, and the cannulae are typically smaller than in high-flow ECMO, bleeding, clotting, and acquired van Willebrand syndrome are nonetheless common complications, influencing the outcome of clinical trials. Of note, hemolysis is one of the major complications, leading to worsening of clinical outcomes and is independently associated with mortality [24–26]. Studies by Braune et al. [5] and Karagiannidis et al. [11] (rotary pumps), as well as del Sorbo et al. [6] (roller pump), demonstrate significant bleeding complications in patients with acute exacerbation of COPD supported with ECCO_2_R. Similar observations were reported in neonatal and pediatric patients [25]. Whereas the complications induced by the oxygenator may be reduced by choosing the most appropriate membrane lung [21], special attention should be given to the blood pumps used at these low blood flow rates. Although blood flow rates may easily be reduced in high-flow ECMO with current rotary pumps, even down to less than 500 mL/min, the flow characteristics change considerably. Rotary blood pumps are developed for a very specific design point, but not for a broad spectrum of blood flow rates from 0 to 8 L/min. The respective components of the pump are dimensioned for this design point to allow for optimal flow guidance, as loss-free and efficient as possible, which may be lost at lower blood flow rates.

An understanding of the capabilities and complications of blood pumps at lower blood flow rates is essential for upcoming clinical trials of ECCO_2_R for patients with ARDS and acute exacerbation of COPD. We therefore sought to investigate the behavior of current ECMO and ECCO_2_R blood pumps with regard to hemocompatibility when operating at low blood flow rates. Since computational fluid dynamics (CFD) has been proven to accurately predict the behavior of blood pumps [27–31], this dedicated method was used to simulate the behavior of three currently used rotary blood pumps across a wide flow range.

## Material and methods

Detailed geometries of the Xenios DP3 (Xenios AG, Heilbronn, Germany), Getinge Rotaflow (Getinge, Gothenburg, Sweden), and LivaNova Revolution (London, UK) pumps were derived from micro-CT scans and manual measurements using computer-aided design. The meshing of the pump’s internal blood volume was determined with tetrahedral elements and refined prism layers at the walls yielding up to 15.2 million mesh elements. Transient result averaging of the simulation results was performed over two impeller revolutions following five revolutions to ensure transient stability. The unsteady Reynolds-averaged Navier-Stokes (RANS) momentum and mass equations were iteratively solved using the commercial element-based finite volume method (ebFVM) solver CFX (ANSYS CFX, ANSYS, Inc., Canonsburg, PA, USA) and the sliding mesh approach. The blood was modeled with a shear-dependent viscosity [32] and a density of 1059 kg m^−3^. Convergence was monitored by the scalar variable residuals and stabilized predictions of the simulation parameters of this study. Detailed information is provided in the online data supplement. To briefly summarized the following.

### Operation range and evaluation parameters

The low blood flow operation ranged between 0.5 and 4 L/min and a lower (150 mmHg) and upper (250 mmHg) pressure head target for typical CO_2_ removal applications. Identical pressure head at a given pump flow was achieved following speed adjustments for each pump (Additional file 3).

### Hydraulic efficiency, secondary flows, and recirculation ratio

The hydraulic efficiency indicates the amount of loss with the conversion of the rotating impeller mechanical energy into hydraulic energy. It is the quotient of hydraulic pump output power to the impeller or shaft power, which can be numerically computed as the product of pump flow rate (*Q*) and pressure rise (*∆P*) and the product of impeller torque (*T*) and angular impeller speed (*ω*). Of note, although the hydraulic efficiency is a useful indicator for the amount of loss during pump operation, a high hydraulic efficiency does not simultaneously imply high hemocompatibility.

1 $$ {\eta}_{\mathrm{hydraulic}}=\frac{P_{\mathrm{Output}}}{P_{\mathrm{impeller}}}; {P}_{\mathrm{Output}}=Q\times \Delta  P,{P}_{\mathrm{impeller}}=T\times \omega . $$

**Fig. 1:**
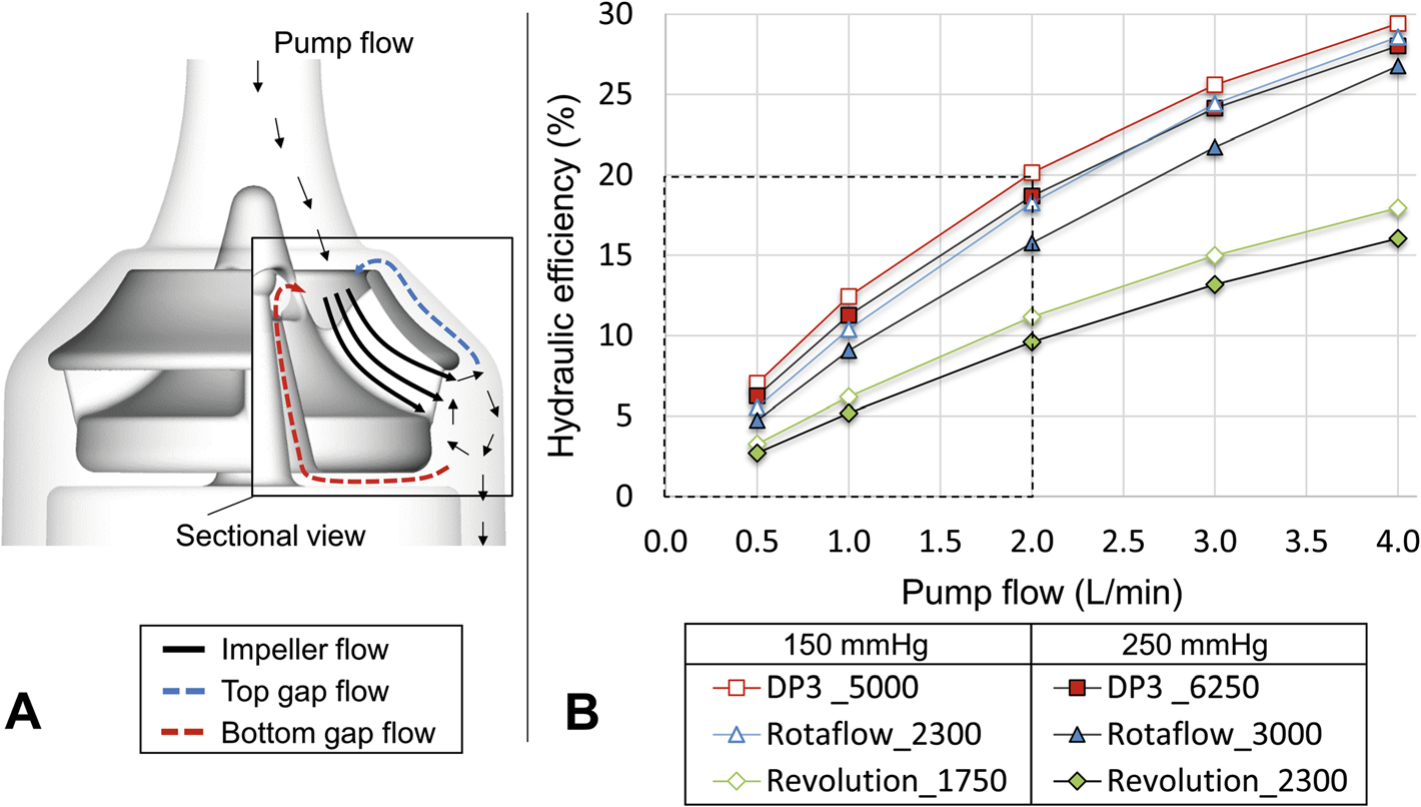
**a** Main (pump flow) and secondary flows and flow paths (top and bottom gap flows) that add up to the impeller flow exemplified using the geometry details of the DP3. **b** Hydraulic efficiency curves of the three blood pumps under study for two constant impeller speeds to realize the pressure head target of 150 mmHg (lower speed in each case) and 250 mmHg

Secondary flows through the gaps between the rotating impeller and stationary housing are essential for adequate washout and to prevent the blood from clotting (Fig. 1a). However, excessive secondary or gap flow leakage can sacrifice the pump’s hydraulic efficiency.

The ratio between all pump internal backflow (also referred to as secondary flow) and pump flow is defined as the recirculation ratio and specifies how often the blood is recirculated within the pump before reaching the pump outlet.

2 $$ {R}_{\mathrm{recirc}}=\frac{\sum {Q}_{\mathrm{secondary}}}{Q_{\mathrm{pump}}\ } $$

### Hemolysis index and shear stress

The hemolysis index, HI (%), describes the percentage of damaged red blood cells with *Δf*Hb as the increase of plasma-free hemoglobin and Hb as the total amount of red blood cells. Current hemolysis estimation models typically relate hemolysis to the scalar shear stress and exposure time *t*_exp_ through a power-law relationship [33]:

3 $$ \mathrm{HI}\left(\%\right)=\frac{\Delta  f\mathrm{Hb}}{\mathrm{Hb}}\times 100=C{t_{\mathrm{exp}}}^{\alpha }{\tau_{\mathrm{scalar}}}^{\beta } $$

The three-dimensional shear stress within the pump was derived from the velocity field obtained from the numerical simulations of the blood flow. It is commonly approximated by a scalar viscous shear stress *τ*_scalar_ following the equation:

4 $$ {\tau}_{\mathrm{scalar}}=\sqrt{2\times {S}_{ij}{S}_{ij}}\times \mu $$

*S*_*ij*_ is the strain rate tensor, and *μ* is the dynamic viscosity of the blood.

The hemolysis index (Eq. 3) was numerically determined for each pump, pump flow, and pressure target employing empirical constants derived for use in rotary blood pumps [31] (C = 1.745 × 10^−6^, *α* = 1.963 and *β* = 0.0762) after conversion to the following equation [34, 35]:

5 $$ \mathrm{HI}={\left(1-\exp \left(-\frac{1}{\dot{Q}\ }{\int}_V{\left(C{\tau}^a\right)}^{\frac{1}{b}} dV\ \right)\right)}^b $$

Of note, numerical blood damage models are under continuous development and cannot fully substitute for experimental hemolysis testing. Nevertheless, numerical hemolysis results show a high correlation with experimental hemolysis results and are a reasonable substitute in the comparative pump analysis of this study.

Platelets of 32 non-septic patients, treated with ECCO_2_R (blood flow rates < 2 L/min) for acute exacerbation of COPD or for ARDS, were retrospectively analyzed in our institution from 2014 to 2018.

## Results

Additional file 1 demonstrates the typical clinical scenario and side effects of ECCO_2_R. Platelets in 32 non-septic patients, treated with ECCO_2_R (blood flow rates < 2 L/min) for acute exacerbation of COPD or for ARDS, dropped by nearly half on average from 242 ± 101 (× 1000/μL) on day 0 to 127 ± 48 (× 1000/μL) on day 13 (Additional file 1A). Additional file 1B demonstrates the typical appearance of clotting within the pump, inducing severe hemolysis as a side effect of the treatment. Three frequently used rotary blood pumps (DP3, Rotaflow, and Revolution) were therefore experimentally evaluated by means of high-resolution CFD.

The hydraulic efficiency of the three blood pumps is demonstrated in Fig. 1. Of note, with decreasing pump flows, all systems present decreasing hydraulic efficiencies towards lower blood flow rates. At 0.5 L/min, the efficiency of the DP3 is only 7% against 150 mmHg of pressure head and 6.2% against 250 mmHg of pressure head; likewise, the hydraulic efficiency of Rotaflow (5.5; 4.7%) and Revolution (3.2; 2.7%) dramatically decreased, barely reaching 12% efficiency at 1 L/min. The DP3 system shows the best hydraulic efficiency at low flows, while the efficiency curves of the Rotaflow show a better trend towards flow rates above 4 L/min.

Higher rotational speeds create an offset towards lower hydraulic efficiency for all systems, meaning that the amount of loss increases.

**Fig. 2:**
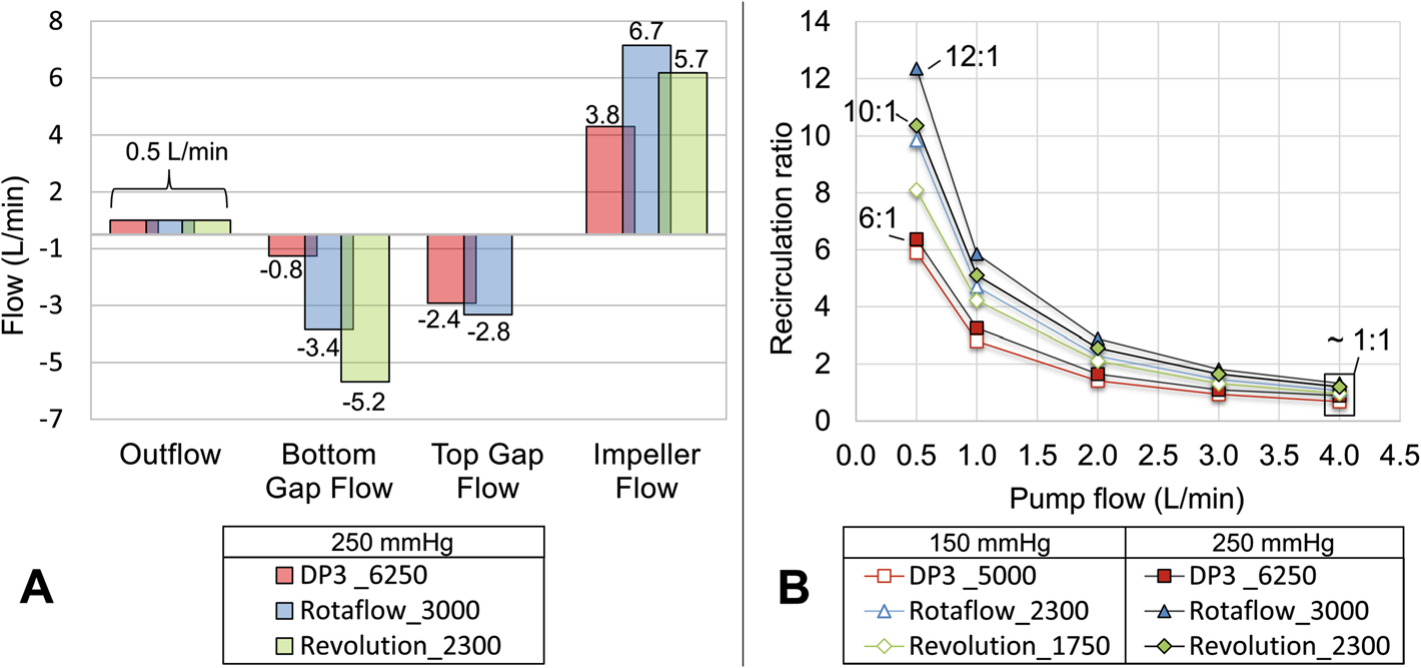
**a** Device-specific secondary gap flows for the high pressure (250 mmHg) and low flow (0.5 L/min) case. The negative sign indicates flow recirculation. **b** Recirculation ratio of the three pump systems for a pressure head of 150 and 250 mmHg

In regard to the recirculation of the blood within the pump, Fig. 2a and b demonstrate the absolute flow rates in the secondary flow gaps in comparison with the impeller flow at 0.5 L/min and 250 mmHg pressure head, and the resulting recirculation ratios respectively. Of note, pumps with suspended rotors characteristically have multiple internal flow paths. The primary or main flow path is designed to generate the pump’s pressure head and fluid flow, while secondary flow paths are required to physically separate rotating impeller components from the stationary ones associated with the casing and to washout necessary gaps and mechanical bearings. Although the pumps effectively pump only 0.5 L/min (main flow), much higher internal backflows exist within the secondary flow paths (Figs. 1a and 2a and Additional file 2). The backflows must be pumped effectively through the impeller in addition to the actual pump flow (main flow), creating very high impeller flows. In Fig. 2b, the ratio between all internal backflow and pump flow is shown by the recirculation ratio (Eq. 2) over pump flow for the low- and high-pressure head target. This ratio becomes increasingly unfavorable for lower pump flows. At 0.5 L/min, it reaches a ratio of 6:1 for the DP3, 10:1 for the Rotaflow, and 12:1 for the Revolution. This means that the blood is likely recirculated between 6 and 12 times within the pumps before reaching the outlet. For higher pump flows (e.g., 4 L/min), this ratio becomes more balanced (0.8–1.2).

**Fig. 3:**
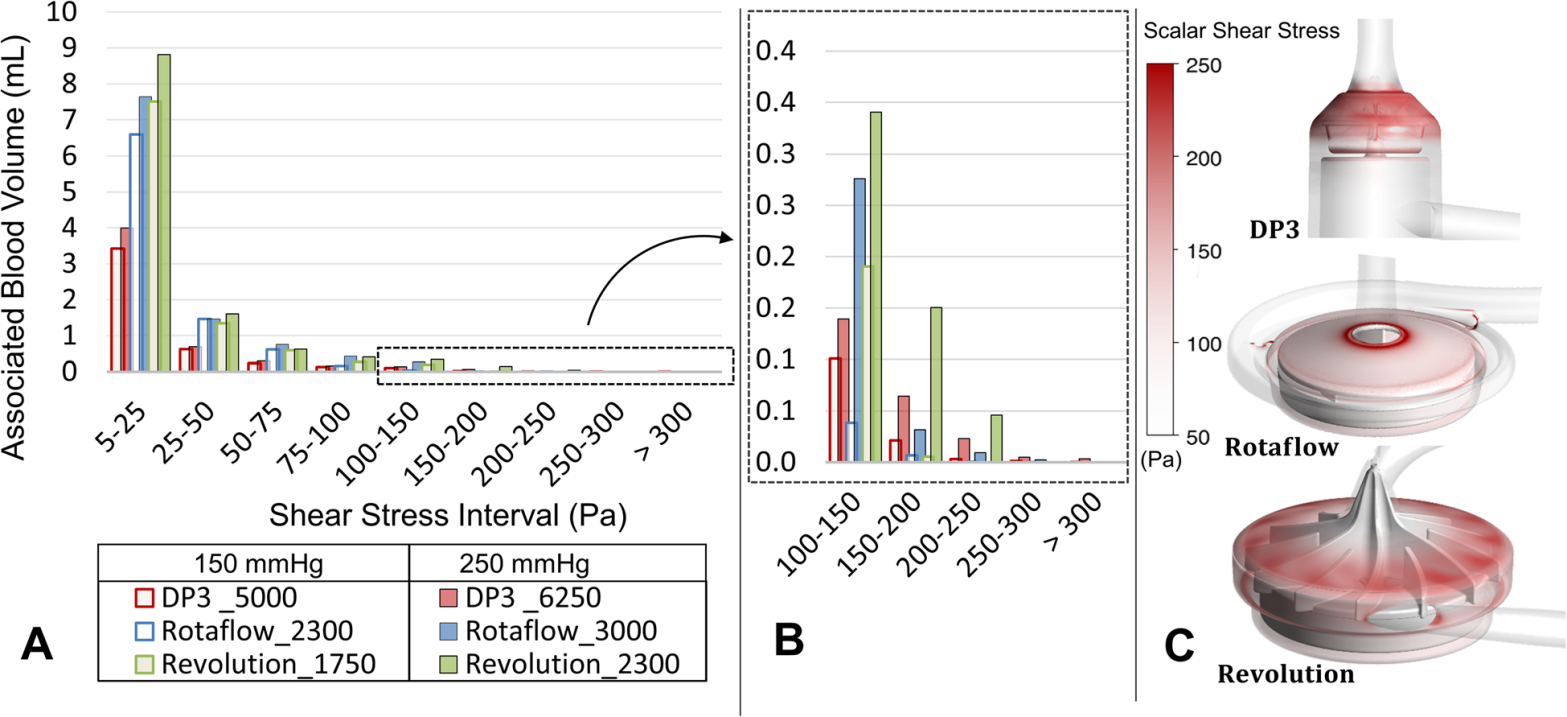
**a** Shear stress histograms for the three pump systems for 0.5 L/min, low- and high-pressure head (150 and 250 mmHg). The blood volume of impeller and secondary gaps associated with a certain shear stress interval (*x*-axis) is plotted (DP3, 9.5 mL; Rotaflow, 18.2 mL; Revolution, 48 mL). The shear stress interval between 0 and 5 Pa contains most of the associated volume and was not shown for an improved view. Figure 4b details the associated volume above 100 Pa. **c** Volume rendering of shear stresses above 50 Pa illustrating potential hotspots within the pumps

Shear stress of blood components is the major side effect generated by rotary blood pumps. Figure 3a depicts the shear stress histograms for all three pumps above 5 Pa. The Revolution (filling volume of 55 mL, largest of the compared pumps) shows consistently higher blood volume distributed over the entire shear stress interval range (Fig. 3a) with particularly more blood volume associated with non-physiological shear stresses above 100 Pa (Fig. 3b). The DP3 (filling volume 18.1 mL) shows more blood volume associated with shear stress regions compared to the Rotaflow (filling volume 28.8 mL). For all three pumps, the associated volume increases with pump speed, which consequently means a redistribution of the blood volume between 0 and 5 Pa to higher shear stress intervals.

**Fig. 4:**
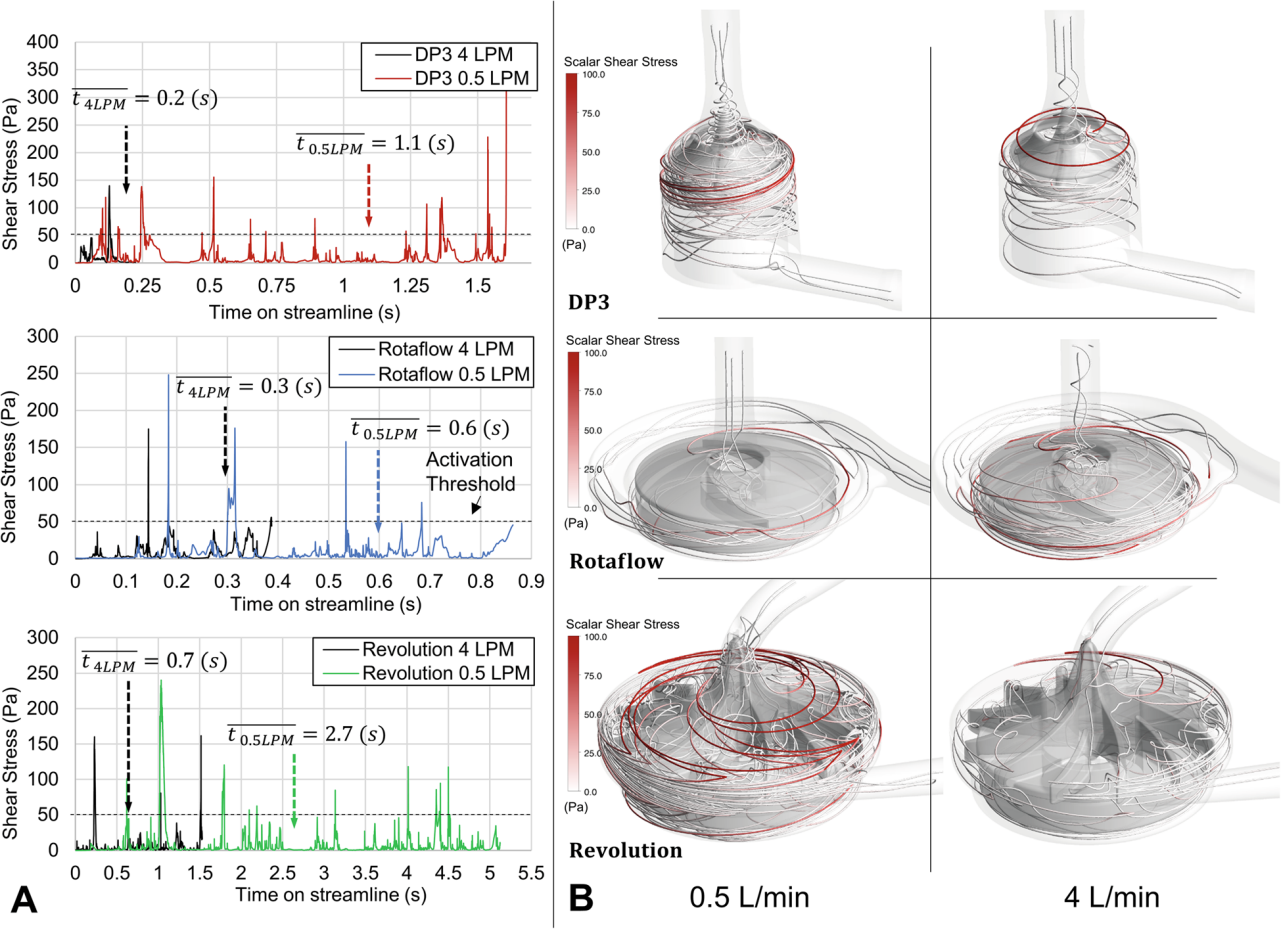
**a** Examples of shear stress profiles along blood streamlines are shown which result from pump flows of 0.5 and 4 L/min. **b** Three representative streamlines and their exposure to shear stress are shown

Representative examples of shear stress profiles along blood streamlines, which result from pump flows of 0.5 and 4 L/min, are shown in Fig. 4. The mean residence times through the pump head were calculated based on 1000 streamlines to provide adequate representation of the complex flow characteristics. Figure 4a and b illustrate how the reduction of the pump flow not only increases the average residence time non-linearly within all pumps, but also causes multiple opportunities for exposure to high shear stresses from the increased internal recirculation (as detailed in Fig. 3), which increase the risk of blood trauma. Hellums [36] showed experimentally that the platelet activation threshold follows a consistent curve over a wide range of conditions on the shear stress-exposure time plane. A platelet activation threshold for blood pumps is conventionally taken as 50 Pa, which corresponds to an estimated particle transit time through the pump of 0.1 s [31]. Higher transit times, as shown in Fig. 4a, might thus condition an even lower activation threshold and thus more platelet activation potential.

**Fig. 5:**
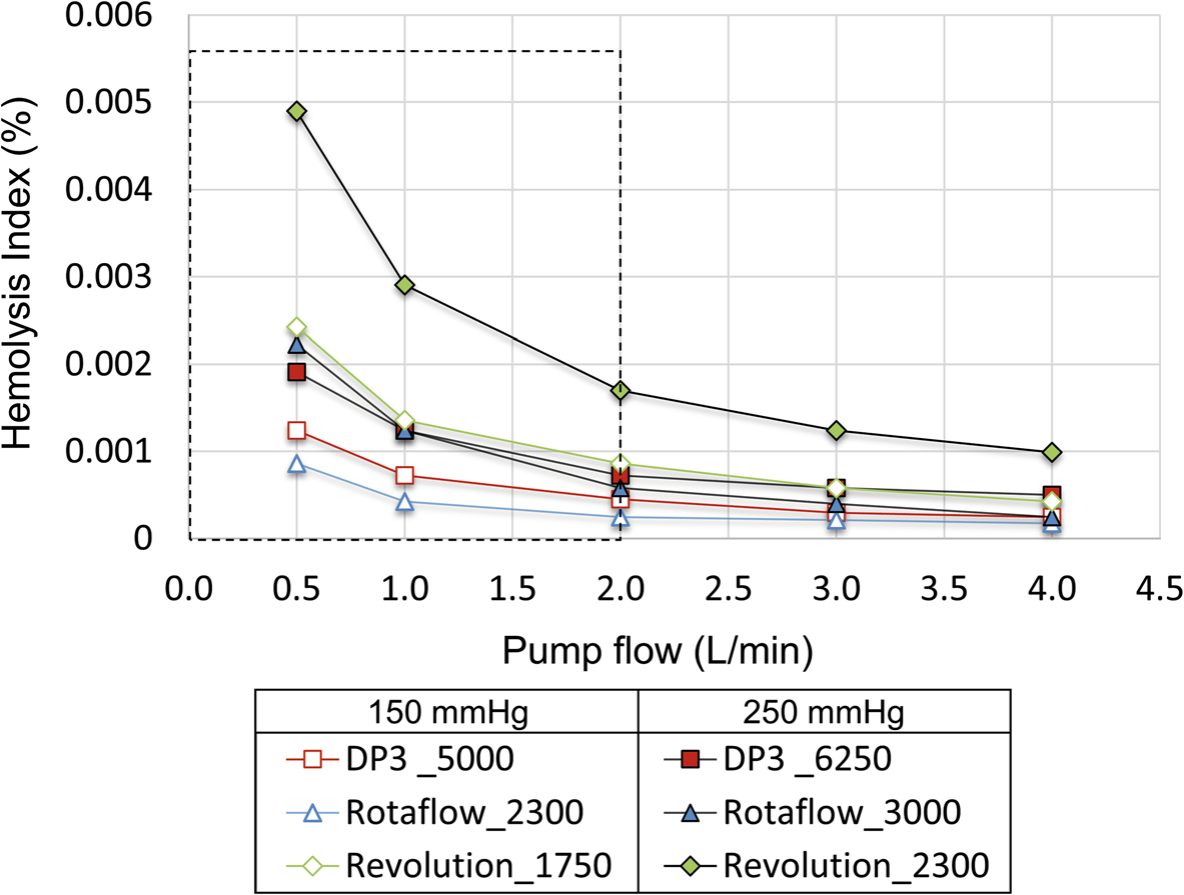
The numerically derived hemolysis index for pump speeds according to the low- and high-pressure head targets (150 and 250 mmHg) and various pump flows

All pump systems show an increase in the hemolysis index (single-pass blood damage) at lower pump flows (Fig. 5). The Revolution appears particularly susceptible to hemolysis compared with the DP3 and the Rotaflow, and the hemolysis index trend towards smaller pump flows is characterized by the largest slope reaching values of approximately 0.005% for 0.5 L/min against 250 mmHg. The curves of DP3 and Rotaflow also increase less steeply, but still significantly, towards smaller pump flows (~ 0.002% for 0.5 L/min against 250 mmHg). Although less blood is pumped through the pump at low blood flow rates, the concentration of damaged blood cells is greatly increased.

## Discussion

For the first time, the present comparative study demonstrates systematically the potentially deleterious effects of currently used rotary blood pumps when operated at blood flow rates below 2 L/min, as is done in the clinical use of ECCO_2_R or neonatal and pediatric ECMO applications. By means of CFD, we could demonstrate that (a) the hydraulic efficiency dramatically decreases to 5–10% if operating at blood flow rates below 1 L/min, (b) the recirculation rate increases 6–12-fold in these flow ranges, and (c) adverse effects are increased due to multiple exposures to high shear stress. The deleterious consequences include a steep increase in hemolysis and destruction of platelets.

The use of ECCO_2_R is rapidly growing, and it remains a promising application of ECLS for ARDS or acute exacerbations of COPD, although there is currently no clear clinical indication for which there is high-quality evidence. Several studies are ongoing or planned for both applications. Although the rationale for the indications is clear, and the prevailing theory is that ECCO_2_R should be safer than ECMO in clinical practice, a concerning number of side effects have been reported in feasibility studies. As an example, major bleeding events occurred in more than 50% of patients in a trial aimed at avoiding invasive mechanical ventilation in patients with acute exacerbations of COPD [5], although this group of patients is not typically prone to bleeding when compared with patients who have severe sepsis. Bleeding may occur from loss of fibrinogen in the setting of its binding to the oxygenator, as well as circuit components, including the blood pumps, affecting the number and function of platelets, as shown in these experiments. Our current data on recirculation, high shear stress, and hemolysis are in line with the observed side effects and are at least in part responsible for this effect. This is of major importance, since, for instance, hemolysis is independently associated with mortality in some groups of patients [25].

From an engineering perspective, operating current blood pumps at low blood flow rates leads to low hydraulic efficiencies aggravating shear stress-induced blood trauma (Figs. 2, 3, and 4). The general efficiency slope of all systems suggests that the maximum efficiency point was designed for higher blood flow rates. Therefore, for all three blood pumps studied, the use of low blood flow rates for ECCO_2_R means this use is considerably removed from the design point of the pumps, meaning the optimal use that the pumps were designed for. The backflows (Fig. 3) must be pumped effectively through the impeller in addition to the actual pump flow, indicating that low pump flow does not also imply low impeller flow. The internal recirculation as presented in Fig. 2 causes multiple exposures to high shear stresses that are not physiologic, especially in the secondary gaps. All secondary flow paths induce fluid flow usually involving low volumetric flow rates and high shear stresses [37]. Given this, the ratio between the main flow and secondary flow at low flow rates might be causally related to the elevated complication risk. All pump systems show an increase of the hemolysis index when operated at blood flow rates below 2 L/min, which is further aggravated below 1 L/min. This is assumed to be a result of (a) the increased residence time of the blood within the pump, in the setting of reducing the pump flow itself and (b) unfavorable internal recirculation (Fig. 2), in combination with (c) multiple exposures to the respective shear stresses (Figs. 3 and 4) of the pump systems considered in this study. The results indicate a fundamental problem of hemocompatibility of all tested pumps for the low-flow operation as used for current ECCO_2_R applications.

Therefore, the concept of ECCO_2_R, which has been proposed as a safer alternative to ECMO due to the lower blood flow rates and smaller cannulae, used is questionable. In fact, the degree of adverse effects attributable to ECCO_2_R in clinical trials has been notably high, belying this notion. The role of blood pumps in contributing to adverse effects at the lower blood flow rates used during ECCO_2_R so far has not been well described. This study demonstrates that, at least in the case of the three pumps studied here, the role is significant. Current rotary blood pumps, such as the DP3, Rotaflow, or Revolution, should be used with caution if operated at blood flow rates below 2 L/min, because of significant and high recirculation, shear stress, and hemolysis.

Hemolysis, platelet function, and bleeding complications should be closely monitored in routine clinical practice and certainly within the context of clinical trials.

### Limitations of the study

Blood damage models are under continuous development and subjected to certain limitations. The strength of current hemolysis models is the qualitative rather than the quantitative analysis. For example, in the context of a high blood recirculation, important correlations such as the cell damage history, which might influence the way a blood cell reacts when exposed to shear stress, are not taken into account. However, numerical predictions and experimentally determined hemolysis results show a very high correlation [38]. Moreover, this study focuses on three frequently used rotary blood pumps. Other rotary pumps or different pump systems (e.g., roller pumps) were not tested and may behave differently. Further experimental hemolysis testing of low pump flows is therefore advised to also illustrate quantitative differences in the hemolytic performance of the pumps considered in this study and other pump systems in general. However, our results are in line with recent data of flow-induced platelet activation, also demonstrating pump thrombogenicity due to long residence time [39].

## Conclusions

The role of blood pumps in contributing to adverse effects at the lower blood flow rates used during ECCO_2_R is shown to be significant in this study. Current rotary blood pumps should be used with caution if operated at blood flow rates below 2 L/min, because of significant and high recirculation, shear stress, and hemolysis. There is a clear and urgent need to design dedicated blood pumps for ECCO_2_R and neonatal/pediatric ECMO applications, which are optimized for blood flow rates in the range of 0.5–1.5 L/min.

## Supplementary information

**Additional file 1. **A: Platelet count trend over 13 days including *n* = 32 non-septic patients. B: Thrombosis formation indicated by arrows in the middle of the pump head.

**Additional file 2.** Geometric representations of the DP3 (a), Rotaflow (b), and Revolution (c). Details of the mesh are provided for the DP3 and Revolution as insets (I + II) for a and b detailing the mesh of the respective gaps between impeller and casing.

**Additional file 3.** Online Data Supplement.

## Data Availability

All data generated or analyzed during this study are included in this published article.

## Abbreviations

CFD: Computational fluid dynamics
HI: Hemolysis index
