# Supplementary figures and images for: Low-flow assessment of current ECMO/ECCO2R rotary blood pumps and the potential effect on hemocompatibility
Source: Crit Care. 2019 Nov 6;23:348. doi: 10.1186/s13054-019-2622-3 (PMC6836552; doi:10.1186/s13054-019-2622-3)

Additional File 2

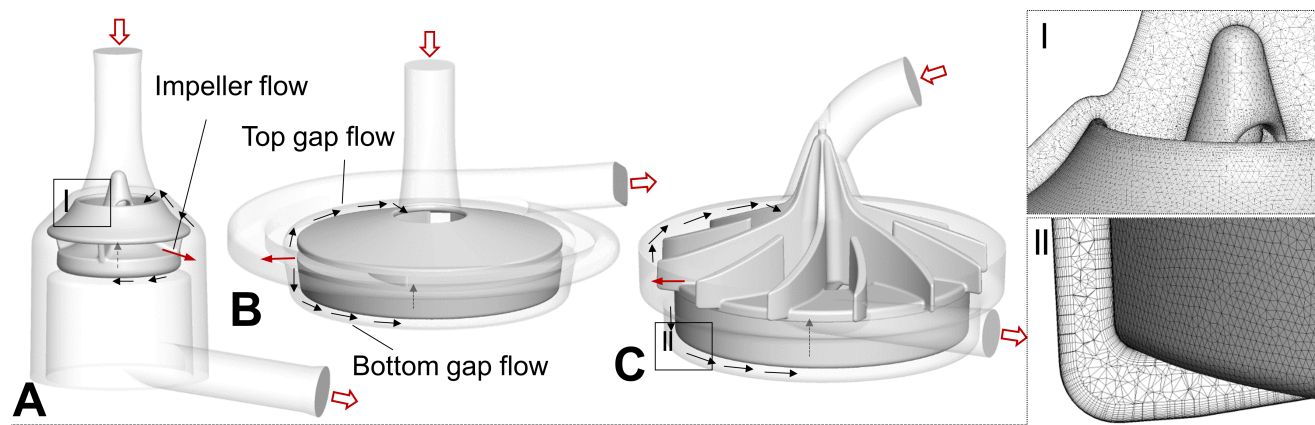

Supplement: Supplementary file 2 — Additional file 2. Geometric representations of the DP3 (a), Rotaflow (b), and Revolution (c). Details of the mesh are provided for the DP3 and Revolution as insets (I + II) for a and b detailing the mesh of the respective gaps between impeller and casing. [file 13054_2019_2622_MOESM2_ESM.pdf]
