# Supplementary material for: Low-flow assessment of current ECMO/ECCO2R rotary blood pumps and the potential effect on hemocompatibility
Source: Crit Care. 2019 Nov 6;23:348. doi: 10.1186/s13054-019-2622-3 (PMC6836552; doi:10.1186/s13054-019-2622-3)
Supplement: Supplementary file 3 — Additional file 3. Online Data Supplement. [file 13054_2019_2622_MOESM3_ESM.docx]

**Additional File 3**

**Supplemental Methods**

Further details of the computational setup and methods

Computer-aided design models were generated using Creo Parametric (PTC Inc, Needham, Massachusetts, USA), resulting in detailed geometries of all three pumps as shown in Supplemental Figure 1. The geometries were meshed with tetrahedral elements and refined prism layers at the walls using the Ansys Mesher (ANSYS Mesher, ANSYS, Inc., Canonsburg, PA, USA), resulting in mesh element numbers between 6.7 and 15.2 Million mesh elements as a result of the vastly different filling volumes of the blood pumps considered in this study, see Supplemental Table 1. The mesh size was automatically reduced for curvatures to optimally conform to the contours of the geometries and to resolve high near-wall velocity gradients, Supplemental Figure 1 I + II. A smooth transition of the prismatic wall elements to the outer mesh was achieved with a first element offset of only 5 µm and up to 18 prismatic wall layers using a prism growth rate of 1.2. The time step for all simulations was proportional to 5° of impeller rotation. In preliminary testing, the influence of time step and mesh size on pressure head, characteristic velocity distribution, and shear stress was determined (1)and the recommended settings resulting in a good compromise between simulation accuracy and computational effort were used for this study.

As a quantitative reliably prediction of blood damage in blood pumps is not yet feasible, the blood damage model (equation 5 of the original manuscript) was employed to allow for a qualitative comparison of the systems, typically providing very high correlation with experimentally derived hemolysis (2).

Equation 5 can be postprocessed by solving a volume integral which results from conversion of equation 3. Details of the conversion and possible limitations are provided in the respective literature (3-5).

**References**

1. Gross-Hardt SH, Boehning F, Steinseifer U, Schmitz-Rode T, Kaufmann T. Mesh sensitivity analysis for quantitative shear stress assessment in blood pumps using computational fluid dynamics. *J Biomech Eng* 2018.

2. Taskin ME, Fraser KH, Zhang T, Wu C, Griffith BP, Wu ZJ. Evaluation of Eulerian and Lagrangian models for hemolysis estimation. *ASAIO J* 2012; 58: 363-372.

3. Garon A, Farinas MI. Fast three-dimensional numerical hemolysis approximation. *Artif Organs* 2004; 28: 1016-1025.

4. Farinas MI, Garon A, Lacasse D, N'Dri D. Asymptotically consistent numerical approximation of hemolysis. *J Biomech Eng* 2006; 128: 688-696.

5. Faghih MM, Sharp MK. On Eulerian versus Lagrangian models of mechanical blood damage and the linearized damage function. *Artif Organs* 2019.

**Supplemental Table 1** Blood filling volume of each pump and corresponding number of necessary mesh elements. **Respective** speed settings for low and high pressure head.

|  | Filling Volume (ml) | Mesh elements (Million) | RPM for 150 mmHg | RPM for 250 mmHg |
| --- | --- | --- | --- | --- |
| DP3 | 18,1 | 6,73 | 5000 | 6250 |
| Rotaflow | 28,8 | 11,45 | 2300 | 3000 |
| Revolution | 55,4 | 15,21 | 1750 | 2300 |
